# Emotional intelligence and perspective-taking as drivers of creativity among mid-level managers in commercial banks in Ecuador

**DOI:** 10.3389/fpsyg.2026.1801171

**Published:** 2026-08-12

**Authors:** Ximena Moscoso, Ruben Guevara

**Affiliations:** CENTRUM Católica Graduate Business School, Pontificia Universidad Católica del Perú, Lima, Peru

**Keywords:** banking sector, creative performance, Ecuador, emotional intelligence, perspective-taking

## Abstract

This study aims to evaluate the relationship between emotional intelligence (EI) and creative performance (CP) in the workplace and their indirect association through perspective-taking (PT). The study applied surveys to 224 mid-level managers from large private banks in Ecuador. The research hypotheses were tested using Partial Least Squares-Structural Equation Modeling (PLS-SEM). The results indicate a significant positive relationship between EI and CP. Furthermore, PT serves as a mediator in that relationship, underscoring the importance of soft skills in fostering workplace creativity. Additionally, the relationships between EI subdimensions and the other variables are presented. This study contributes to the literature on EI and CP by identifying PT as a key interpersonal cognitive mechanism through which EI translates into CP. By positioning PT as the cognitive bridge linking EI and CP, this study offers a more nuanced explanation of the underlying psychological processes that connect emotional competencies with creativity, thereby extending both the emotional intelligence and creativity literatures, confirming the complementary explanation of this process by the Ability Model of EI, the social cognitive theory, and the dynamic componential theory of creativity. The theoretical implication is that integrating these theories into a coherent causal framework offers a more robust explanation of CP than considering each theory in isolation. From a practical standpoint, these findings suggest that organizations seeking to enhance CP should invest not only in enhancing employees’ EI but also in cultivating PT as a critical interpersonal capability. Finally, the study introduces a new structural equation model with moderate explanatory and medium predictive power.

## 1 Introduction

The world of business faces intense competition, rapid technological changes, and relatively short product life cycles, which challenge organizations to remain competitive (Ahmad et al., 2024; Rietzschel et al., 2024). Creativity and innovation are essential organizational strengths (Ciby and Dominic, 2026), especially in the service industry (Salim and Khorsheed, 2025). The most creative companies gain a competitive edge (Skavronska, 2023). Amabile and Pratt (2016) suggested that creativity and innovation are part of the same process and key competencies for organizations, driving social and organizational benefits, particularly in Latin American countries with unstable markets (Zapata-Cantu and González, 2021) and a large number of small and medium-sized enterprises, where creativity may also contribute to happiness at work (Martínez-Arvizu et al., 2025).

Innovation is the successful implementation of creative ideas (Amabile and Pratt, 2016), and creative employees are foundational to innovation (Chen et al., 2021). As companies demand initiative and creative thinking from employees (McKay et al., 2024), scholars have focused on understanding workplace creativity (Christensen-Salem et al., 2021; Ishiguro et al., 2026; Le and Phan, 2024). Despite growing interest in workplace creativity, important gaps remain in understanding the factors that shape creative performance (Abdulaziz and Sulphey, 2026), particularly the role of individual psychological traits and motivational mechanisms (Darvishmotevali et al., 2018; Zhao et al., 2023). Existing literature shows a limited incorporation of motivation-related constructs and other psychological dimensions within creativity research, suggesting that these variables remain underexplored despite their potential influence on employees’ creative outcomes (Panda and Swamy, 2025). At the same time, the emergence of malevolent creativity as a research stream has raised concerns about the potential negative consequences of creativity when directed toward harmful or unethical purposes (Fousiani et al., 2025; Xing et al., 2024; Zhou et al., 2023). This highlights the need to identify mechanisms that not only foster creativity, but also channel it toward constructive and socially beneficial outcomes. Although prior studies have emphasized the importance of enhancing creative performance in organizations (Gong et al., 2018), limited research has examined how motivational and psychological factors can simultaneously promote positive creativity while mitigating malevolent creativity, particularly in underrepresented contexts such as Latin American countries where possible products of malevolent creativity, such as corruption and illegal economies, are a growing concern (Dutra de Paulo et al., 2022). Consequently, further research is needed to develop a more comprehensive understanding of the psychological drivers of workplace creativity and their implications for fostering ethical and positive creative performance in Latin American organizational settings.

This study adopts the term creative performance as a proxy for creativity at work (Koseoglu et al., 2022; Zhao et al., 2023). CP has been explored in relation to personal traits as promotors, including emotional elements (Ivcevic and Brackett, 2015). Research suggests EI predicts work-related outcomes (Prentice et al., 2019), but links between EI and CP are not yet clear (Friedrich et al., 2025) and potential mediators need consideration (Sapiee et al., 2024). Additionally, Schlaegel et al. (2022) reported that most prior studies examined overall EI rather than using its specific dimensions, which limited specific findings from EI dimensions. Furthermore, our literature review indicates this is particularly true in the organizational realm. On the other hand, Salminen et al. (2021) suggested that EI aids ideation, in which client perspectives are crucial. Organizations can benefit when their employees understand others’ viewpoints. PT is rarely studied in organizations (Calvard et al., 2023). Sharma and Tiwari (2024) found that most EI studies were conducted in Western countries, underscoring the need to examine EI in other contexts to account for cultural nuances that may affect outcomes.

Perspective-taking (PT) is a proximal interpersonal cognitive mechanism because it involves actively simulating and integrating others’ viewpoints, thereby reshaping individuals’ cognitive representations of social and problem-solving situations. Research in social cognition shows that PT requires decentering from one’s own viewpoint and constructing alternative mental models, enhancing cognitive flexibility and the integration of diverse information (Ames, 2004; Davis, 1983). Consistent with this, creativity research indicates that PT facilitates divergent thinking and idea recombination by broadening attentional scope and reducing cognitive fixation (Grant and Berry, 2011; Hoever et al., 2012). Thus, it operates directly on the cognitive processes underlying idea generation. Therefore, PT is distinct in that it captures the cognitive transformation of social information into alternative mental representations, making it a more proximal mechanism linking interpersonal experience to creative cognition.

Higher levels of perspective-taking are expected in collectivist cultures. Ecuador is known for its strong collectivist culture and, according to Chopik et al. (2017), it registers the highest perspective-taking score globally. Motivated by the preceding arguments, this research was conducted in Ecuador; the objective was to analyze the relationship between emotional intelligence (EI) and its specific subdimensions and creative performance (CP). The study specifically sought to evaluate the mediating role of perspective-taking (PT) within the EI-CP relationship, offering fresh theoretical perspectives on how emotional processes facilitate the generation of novel ideas.

## 2 Theoretical framework and hypothesis development

Bandura’s social cognitive theory (SCT) provides a comprehensive, holistic framework for this study, as it connects emotional and cognitive processes with behavior. It accommodates both individual (self-regulation and self-efficacy) and social (perspective-taking) components (Bandura, 1986). This general theory is complemented in this study with the dynamic componential theory of creativity (DCTC), which posits that creativity emerges from the dynamic interaction of domain-relevant skills, creativity-relevant processes, intrinsic motivation, and the social work environment, with these components influencing creative performance over time rather than remaining static. The theory emphasizes that intrinsic motivation and contextual factors can enhance or constrain creativity, making creative output the result of an evolving person–environment system rather than solely individual traits (Amabile, 1983, 1996). The dynamic extension further explains how affect, motivation, and organizational context continuous behavior shape the creative process and outcomes (Amabile and Kramer, 2011; Amabile and Pratt, 2016), and the ability model of EI (AMEI), which conceptualizes emotional intelligence as a set of cognitive abilities involving the perception, use, understanding, and regulation of emotions to facilitate adaptive thinking and behavior, rather than as a personality trait (Mayer and Salovey, 1997; Salovey and Mayer, 1990). This model proposes that these emotion-related abilities can be measured as a form of intelligence and contribute to effective reasoning, decision-making, and social functioning (Mayer et al., 2004).

### 2.1 Emotional intelligence

Mayer and Salovey (1997) affirmed that early in the last century, three types of intelligence were acknowledged: verbal-positional intelligence, spatial-performance intelligence, and social intelligence, but this third concept could not be developed because of its high correlation with the other two, thereby emerging the concept of emotional intelligence to replace that of social intelligence. EI is a set of cognitive abilities involving the perception, use, understanding, and regulation of emotions to facilitate adaptive thinking and behavior, rather than as a personality trait (Mayer and Salovey, 1997; Salovey and Mayer, 1990). Furthermore, Mayer et al. (1999) argued that EI is broader than social intelligence because, in addition to reasoning about emotions in social contexts, it includes reasoning about internal emotions, thereby facilitating personal growth. They also held that EI gathers the necessary features to be considered as a type of intelligence, because: (a) it is a mental ability, (b) it manifests specific correlational patterns among itself and other types of intelligence, and (c) it develops with age and experience.

Regarding theoretical frameworks of emotional intelligence, Rooy and Van Viswesvaran (2004) identified two main models: the ability model, proposed by Mayer and Salovey (1997), and the mixed models, developed by Goleman (1995) and Bar-On (1997). The ability model emphasizes the interplay between emotion and cognition and posits that EI is dependent on general mental ability (GMA; Mayer et al., 1999). In contrast, mixed models incorporate broader constructs, such as personality traits, and therefore consider the link between EI and GMA to be less significant.

This study adopts the ability-based conceptualization of EI, as previous research has recognized its stronger theoretical foundation compared with mixed approaches (Daus and Ashkanasy, 2005; Joseph and Newman, 2010; Prentice et al., 2019). Accordingly, the research examines EI as a multidimensional construct encompassing four components: self-emotional appraisal (SEA), others’ emotional appraisal (OEA), regulation of emotions (ROE), and use of emotions (UOE; Wong and Law, 2002).

### 2.2 Creative performance

According to the DCTC, creativity emerges from the dynamic interaction of domain-relevant skills, creativity-relevant processes, intrinsic motivation, and the social work environment, with these components influencing creative performance over time rather than remaining static. Employees’ CP has consistently been highlighted in academic and practitioner research as an essential factor in performance (Sahadev et al., 2024). Even though ideas may occasionally arise spontaneously, CP demands intentional effort. In this process, cognitive elements are essential and usually closely connected with emotional and behavioral components (Zielińska et al., 2024). The social environment is also important, as coworkers’ daily attitudes, through one-on-one interactions or group dynamics, may significantly affect employees’ creativity (Amabile and Pratt, 2016). Work loneliness is detrimental to CP (Firoz and Chaudhary, 2022) whereas colleagues can act as catalysts to enhance others’ CP (Koseoglu et al., 2022).

### 2.3 Perspective taking

Perspective-taking is defined as “the active cognitive process of imagining the world from another’s vantage point or imagining oneself in another’s shoes to understand their visual viewpoint, thoughts, motivations, intentions, and/or emotions.” (Ku et al., 2015, pp.94–95). It is often subdivided into cognitive and affective components. The cognitive aspect refers to the capacity to understand another person’s thoughts or beliefs, whereas the affective aspect involves recognizing or inferring another person’s emotions or feelings (Healey and Grossman, 2018).

Gehlbach (2004) claimed that PT is a concept comprising a cognitive ability and a motivational component, reflected in the propensity to take others’ perspectives. Additionally, cultural differences may lead to different patterns of PT; a culture that emphasizes interdependence focuses attention on others, while a culture that emphasizes independence focuses attention on the self (Wu and Keysar, 2007).

Furthermore, SCT (Bandura, 1986) posits that individuals actively acquire and process information through reciprocal interactions with their social environment. Through observational learning and self-regulatory processes, individuals interpret others’ behaviors, intentions, and emotional cues, thereby adapting their responses to social situations. In this context, perspective-taking represents an important interpersonal cognitive process through which individuals understand and integrate others’ viewpoints, thereby facilitating effective social interaction and collaborative problem-solving.

### 2.4 Emotional intelligence and perspective-taking

Emotions and perspective-taking are integral to nearly all forms of social interaction, yet the mechanisms linking these two processes remain insufficiently understood. Both the cognitive and affective components of perspective-taking (PT) rely on emotional foundations, although this reliance is less pronounced in cognitive PT (Healey and Grossman, 2018). Emotional states influence the cognitive mechanisms underlying PT, as the degree of self-prioritization during perspective-taking varies with an individual’s emotional state (Bukowski et al., 2015). In this regard, prior research suggests that negative emotions are associated with heightened self-focus (Mor and Winquist, 2002).

Empirical evidence indicates a positive relationship between emotional intelligence (EI) and PT (Pongrac et al., 2019; Shi and Du, 2020). This association may arise because adopting and identifying with another’s perspective inherently evokes a range of emotional responses (Brügge-Feldhake et al., 2024). Individuals with higher EI tend to exhibit greater verbal and social intelligence (Mayer et al., 2009), whereas those with lower EI often experience difficulties forming meaningful social connections (Brackett and Salovey, 2006). Consequently, emotionally intelligent individuals are more likely to adopt others’ perspectives, whereas those with low EI may experience discomfort in social contexts or communication challenges that hinder PT (Shi and Du, 2020).

Emotional competencies thus provide individuals with both the capacity for self-reflection and the ability to empathize with others (Schneider et al., 2021). Moreover, EI can predict relationship quality, as emotions convey information that fosters empathic connections between individuals (Caruso et al., 2015).

The relationship between EI dimensions and PT has been explored, revealing positive associations among attention to feelings, clarity of emotions, and mood repair and perspective-taking (Fernández-Abascal and Martín-Díaz, 2019; Gómez-Leal et al., 2021). However, research on the specific associations between self-emotional appraisal (SEA), others’ emotional appraisal (OEA), and use of emotion (UOE) with PT remains limited. In contrast, the relationship between regulation of emotion (ROE) and PT has received comparatively greater attention.

Theoretical and empirical literature suggests that SEA and PT are connected through appraisal-based mechanisms and self-simulation processes. Evaluating one’s own emotions may either facilitate or bias perspective-taking. Epley et al. (2004) proposed that individuals adopt others’ perspectives by adjusting from their own using the self as a reference point or “template” in the process. Similarly, Chambers and Davis (2012) argued that people gauge how much empathy to feel by imagining themselves in another’s situation, showing greater empathy when such self-projection is easy and less when it is difficult. Supporting this view, Rieffe and Camodeca (2016) found that individuals who attend closely to both their own and others’ emotions demonstrate more sophisticated empathic abilities, encompassing both the affective and cognitive dimensions of empathy.

Olderbak and Wilhelm (2017) found that emotion perception was moderately related to PT. Emotion perception represents a fundamental cognitive process that involves recognizing emotional cues in the environment, such as facial expressions or tone of voice. In contrast, cognitive empathy is a more advanced process that requires individuals to infer others’ intentions, beliefs, and mental states. Neuroscientific evidence further supports this conceptual connection: studies have identified overlapping yet distinct neural substrates underlying emotion perception and cognitive empathy, suggesting a functional interrelation between the two (Mitchell and Phillips, 2015).

Conversely, difficulties in emotion appraisal may impair automatic perspective-taking. Rodriguez et al. (2022) observed that reduced emotional awareness -manifested as lower attention and higher impulsivity- was linked to poorer performance in tasks requiring automatic recognition of others’ perspectives. This suggests that emotional insight plays a critical role in facilitating spontaneous social understanding.

Furthermore, several studies have confirmed a positive association between emotion regulation (ROE) and PT. Salazar Kämpf et al. (2023) found that individuals who regulate their emotions adaptively are better able to adopt others’ perspectives. In contrast, those who rely on maladaptive strategies such as suppression or rumination are more self-focused, thereby hindering PT. Thompson et al. (2024), further, corroborated this relationship, suggesting that effective emotion regulation supports cognitive empathy by enabling individuals to manage their own affective responses and thus engage more constructively with others’ emotional states. In contrast, individuals characterized by low attentional control and high impulsivity -traits associated with poor emotion regulation- may require more time to establish their own perspective, yet tend to rush when adopting another’s viewpoint (Rodriguez et al., 2022).

On the other hand, the SCT and the AMEI complement each other to support the relationship between EI and perspective-taking. The AMEI proposes that individuals differ in their capacity to perceive, use, understand, and regulate emotions (Mayer and Salovey, 1997; Salovey and Mayer, 1990). These emotional abilities enhance individuals’ capacity to interpret others’ emotional states accurately and to regulate their own affective responses, thereby facilitating perspective-taking. Consistent with the SCT (Bandura, 1986), emotionally intelligent individuals are better equipped to process social information, exercise self-regulation, and adapt their behavior during interpersonal interactions, making perspective-taking a natural consequence of higher emotional intelligence. As a consequence, the proposed hypotheses are:

Hypothesis 1 (H1): Emotional intelligence positively relates to perspective-taking.

Hypothesis 1a (H1a): Self-emotional appraisal positively relates to perspective-taking.

Hypothesis 1b (H1b): Others’ emotional appraisal positively relates to perspective-taking.

Hypothesis 1c (H1c): Use of emotions positively relates to perspective-taking.

Hypothesis 1d (H1d): Regulation of emotions positively relates to perspective-taking.

### 2.5 Perspective-taking and creative performance

Creativity is inherently a social phenomenon, with interactions among individuals significantly shaping the creative processes within organizations (Dhir and Vallabh, 2025; Kung and Chao, 2019). Perspective-taking (PT) emerges as a critical relational skill that facilitates navigation through the social dynamics integral to creativity (Ng et al., 2021). Empirical evidence indicates that learning from others enhances creative performance (Ma et al., 2023). Similarly, teamwork plays an essential role in fostering individual creativity, particularly in organizations operating in service-oriented sectors (Kulichyova et al., 2025). Even employees with narcissistic traits tend to boost their creative self-efficacy - and ultimately their creativity- when they see coworkers engaging in creative behaviors (Guo, 2025). Likewise, the development of meaningful work–work that benefits others–and intrinsic motivation are pivotal drivers of creativity, as intrinsically motivated employees concentrate their cognitive resources more effectively to generate novel outcomes (Amabile and Pratt, 2016; Liu et al., 2016). PT enables individuals to understand others’ needs, thereby fostering purposeful efforts to devise creative solutions.

Within the framework of SCT, PT aligns with social learning concepts, in which individuals acquire behavioral patterns by observing others’ perspectives. This process promotes collaboration and social interaction, which are conducive to creative endeavors. Consequently, adopting multiple perspectives is recognized as an effective strategy for generating original ideas (Chou and Tversky, 2020). Additionally, PT enables individuals to integrate others’ viewpoints, expanding the information available for problem solving. Consistent with the SCT (Bandura, 1986), perspective-taking enriches social information processing through observational learning and social interaction. Moreover, the DCTC (Amabile, 1996; Amabile and Kramer, 2011) suggests that integrating diverse perspectives enhances creativity-relevant cognitive processes, increasing the likelihood of CP. Employees with high PT capacity consider diverse viewpoints and demonstrate superior performance on divergent thinking tasks (Anderson et al., 2023; Shukla and Kark, 2020). This dual capacity fosters both the exploration and harmonization of ideas, enhancing the refinement of creative outputs and facilitating acceptance among peers. Moreover, empathizing with customers by adopting their viewpoint is essential for creating products or services that effectively satisfy user needs (Nadiia et al., 2019). Attending to others’ perspectives enables employees to acquire a holistic understanding of valuable ideas for colleagues, supervisors, clients, and other stakeholders who evaluate the benefits of their work (Grant and Berry, 2011).

Multiple studies have demonstrated robust associations between PT and creative performance (Han et al., 2021; Hu and Luo, 2020; Li and Liao, 2017). Engaging with alternative viewpoints can trigger cognitive reappraisal processes that integrate diverse perspectives and ideas, thereby enhancing creativity (Hargadon and Bechky, 2006). Thus, PT serves as a mechanism to leverage collective team cognition and amplify individual creativity. Hoever et al. (2012) further identified PT as a cognitive mediator that explains the positive impact of feedback on creative performance, as it simultaneously acknowledges recipients’ viewpoints and cultivates a supportive environment.

Creative ideas must embody both novelty and utility. PT behaviors during the creative process expose individuals to a breadth of opinions and viewpoints, which constitute foundational prerequisites for creativity (Li and Liao, 2017). Individuals employing PT tend to generate ideas with greater potential to influence solutions at broader analytical levels (Litchfield et al., 2014). Rubenstein et al. (2019) found that PT was strongly correlated with the selection of original and useful ideas and their elaboration. Moreover, PT emerged as the most significant predictor of creative fluency and showed significant associations with creative flexibility.

This synthesis underscores PT as a multifaceted cognitive and social skill instrumental in fostering creativity through enhanced perspective integration, collaboration, and motivational mechanisms.

Thus, the following hypothesis is:

Hypothesis 2 (H2): Perspective-taking positively relates to creative performance.

### 2.6 Emotional intelligence and creative performance

Social cognitive theory highlights the importance of self-regulation, which is closely tied to EI, as it involves recognizing and managing one’s emotions, which is essential for problem-solving and creativity. Employees with elevated EI can better regulate their responses to challenges, contributing to higher creativity. Individuals can harness their emotions to guide thoughts toward problem-solving, evoke emotions to enhance task performance, and regulate emotions to maintain interest and effort. All of this is crucial for overcoming difficulties in the creative process (Ivcevic and Hoffmann, 2019). The DCTC emphasizes the importance of emotions and social interaction for creativity and suggests that different emotions may play different roles at different stages of the creative process. For example, positive emotions are positively associated with task-related and interactional learning, both of which boost employee creativity (Liu et al., 2022). Individuals with high levels of EI evidence sharp creative capabilities within professional domains (Kasuma and Rusdi, 2024). EI explains variation in creative behaviors because organizational members who use and regulate their emotions appropriately foster a trusting atmosphere, thereby facilitating idea sharing and motivating creative potential (Carmeli et al., 2013).

Salovey and Mayer (1990) held that people who can appraise their own emotions may better control frustration and more easily confront change, which, according to Bozionelos and Singh (2017), facilitates employees’ collaboration and knowledge sharing with colleagues. Likewise, ROE may transform negative and ambivalent emotions into creativity promoters (Leung et al., 2021). Similarly, and especially relevant in this era of constant distraction, a study on Generation Z found that ROE can protect cognitive performance from the anxiety caused by disconnection from digital networks, thereby fostering creativity (Krisnanda et al., 2026). Emotionally intelligent people are more likely to control and manage their negative emotions (Kundi and Badar, 2021) so that feelings of tension or conflict, for example, might facilitate integrative complex thinking and promote the generation of creative ideas (Leung et al., 2018). Anger has also been identified as highly correlated with CP (Xing et al., 2024). In the same way, mixed interpersonal emotions may positively affect collective creativity by creating an appropriate space to identify connections between unrelated ideas (Kung and Chao, 2019).

Finally, literature presents several studies reporting a positive relationship between emotional intelligence and the creative performance of employees in recent years (Fujii, 2025; Kasuma and Rusdi, 2024; Stawicki et al., 2022).

Therefore, the following hypotheses are proposed:

Hypothesis 3 (H3): Emotional intelligence positively relates to creative performance.

Hypothesis 3a (H3a): Self-emotional appraisal positively relates to creative performance.

Hypothesis 3b (H3b): Others’ emotional appraisal positively relates to creative performance.

Hypothesis 3c (H3c): Use of emotions positively relates to creative performance.

Hypothesis 3d (H3d): Regulation of emotions positively relates to creative performance.

### 2.7 Emotional intelligence, perspetive-taking and creative performance

Social cognitive theory emphasizes the dynamic interplay between cognitive, emotional, and behavioral factors. EI can be seen as a key cognitive-emotional resource that shapes CP. It aligns with the idea that emotions and cognitive processes (such as perspective-taking) influence how one engages with tasks and others in the workplace (Bandura, 1986). PT can be viewed as a mediator that enhances social learning and problem-solving, thereby boosting creative performance.

Perspective-taking has been addressed as a mediator in several studies. Fasbender et al. (2023) examined PT as a mediator between coworker well-being and the focal employee’s well-being. They concluded that understanding and considering coworkers’ perspectives indirectly improved well-being through the support they received. However, this same practice negatively affected employees’ well-being due to the mental exhaustion caused by perspective-taking. Li and Liao (2017) examined how help-giving influences employee creativity, with the mediating effect of PT. Their findings indicated that offering assistance during creative problem-solving fosters PT, thereby increasing creativity. The mediating effect of PT has been confirmed again in the relationship between leader practices and employees’ creativity; thus, PT acts as a mediator between leader humility and employees’ cognitive appraisal of creativity, even in a crisis (Wang et al., 2017). The same effect was found between transformational leadership and CP (Wadei et al., 2021). These findings suggest that employees with high levels of PT can absorb input from their superiors and channel it into creativity.

Hui et al. (2022) studied the moderating effect of PT between moral reasoning and creativity. Their results indicated that individuals who lacked PT skills often exhibited lower levels of moral reasoning, especially if they were also creative. Consequently, PT may contribute to the production of creative outputs designed under moral reasoning, mitigating the emergence of a malevolent facet of creativity that may appear when emotions such as anger are present (Xing et al., 2024).

The literature acknowledges that attention and repair of emotions predict PT, (Hodgson and Wertheim, 2007) and considering others’ perspectives helps generate more ideas and promotes more constructive judgments of those ideas (Wang et al., 2017) Consequently, it is expected that the ability to perceive, regulate, and use emotions will affect creativity through the capacity to put oneself in another’s situation. According to Salminen et al. (2021), “emotional intelligence may be beneficial in the ideation process where the perspective of the customer or end user has to be considered” (p. 191).

Finally, it is important to note that mediators such as intrinsic motivation, creative self-efficacy, knowledge sharing, and psychological empowerment are more distal motivational, behavioral, or contextual states. Intrinsic motivation reflects an energizing affective condition that drives engagement in creative tasks rather than explaining how ideas are generated (Amabile, 1996). Creative self-efficacy captures beliefs about creative capability (Tierney and Farmer, 2002); knowledge sharing reflects information-exchange behavior (Wang and Noe, 2010); and psychological empowerment reflects perceived autonomy and meaning (Spreitzer, 1995). While all influence creativity, they operate upstream of cognition rather than directly explaining cognitive transformation.

Based on these considerations, the next hypotheses are proposed:

Hypothesis 4 (H4): Perspective-taking mediates the relationship between emotional intelligence and creative performance.

Hypothesis 4a (H4a): Perspective-taking mediates the relationship between self-emotional appraisal and creative performance.

Hypothesis 4b (H4b): Perspective-taking mediates the relationship between others’ emotional appraisal and creative performance.

Hypothesis 4c (H4c): Perspective-taking mediates the relationship between use of emotions and creative performance.

**FIGURE 1:**
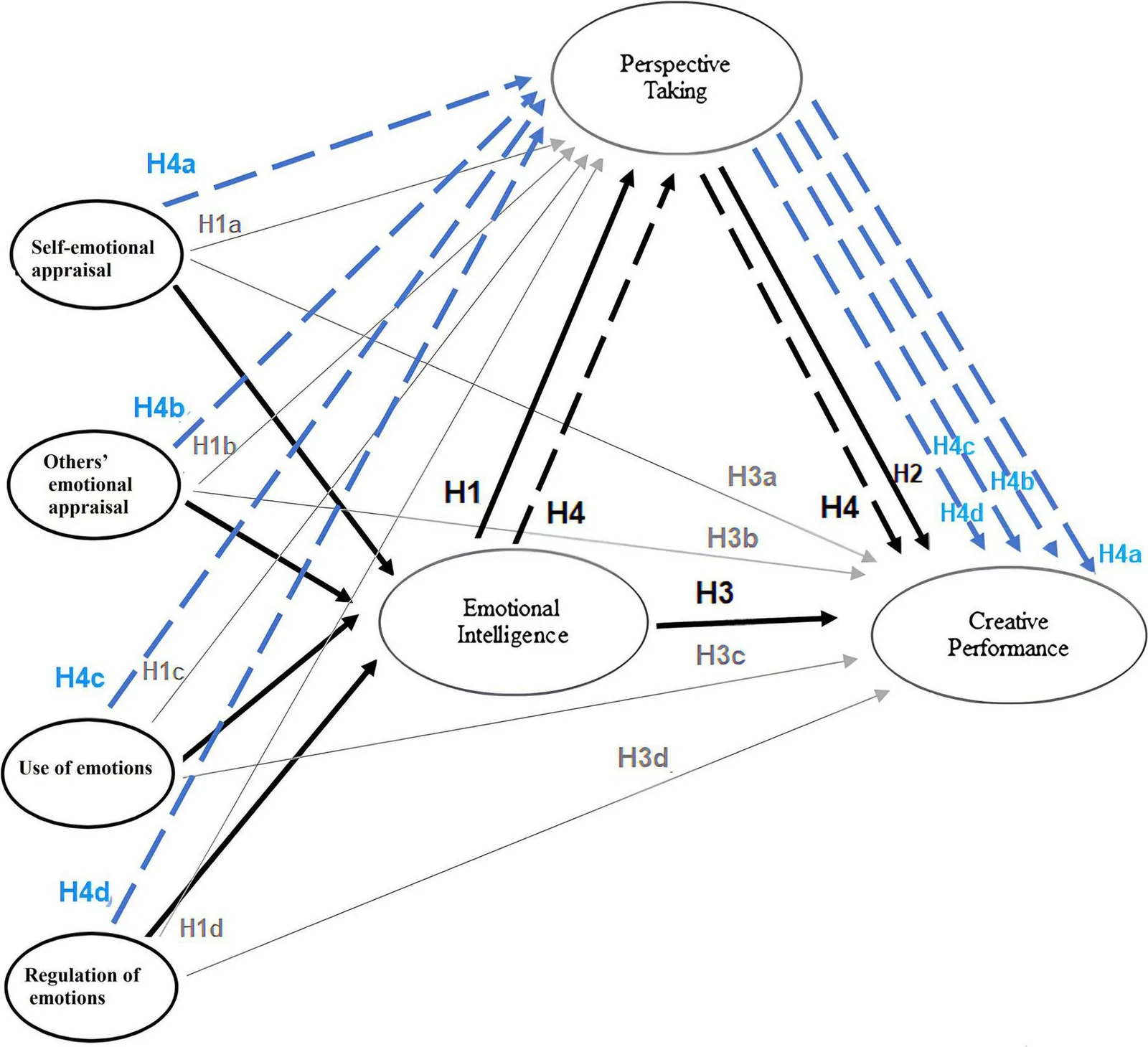
Proposed conceptual model.

Hypothesis 4d (H4d): Perspective-taking mediates the relationship between regulation of emotions and creative performance. Figure 1 shows the proposed conceptual model for this study.

## 3 Materials and methods

This research followed a non-experimental, cross-sectional, correlational, and explanatory design. Data collection occurred at a single time point, and no variables were manipulated. This research utilizes an individual-level analysis as each construct was theoretically defined and empirically quantified at the level of the individual respondent. The study aimed not only to assess the associations among the variables but also to explain the underlying pathways through which these relationships may occur.

### 3.1 Population

The target population of this study comprised mid-level managers working in private commercial banks in Ecuador. Participants were included according to specific criteria that reflect the characteristics of the population (Babakus et al., 2017) such as having at least 6 months of managerial experience. According to Superintendencia de Bancos (2023), 24 private commercial banks comprise the Ecuadorian financial system. The population was limited to large banks with the largest market share and an AAA rating, indicating their solvency, risk management capacity, and fulfillment of public obligations (Superintendencia de Bancos, 2024). Four out of 24 banks were classified as large banks at the time of the survey, and three of these agreed to participate. The study involved the head local or regional offices of these banks. A census was used, enabling a complete representation of the chosen population (Burakauskaitė and Čiginas, 2023).

### 3.2 Research instruments

Emotional intelligence was measured using the Wong and Law (2002) Emotional Intelligence Scale (WLEIS), developed for organizational research. It consisted of 16 items across four dimensions, with four items per dimension, e.g., SEA: “I have good understanding of my own emotions”; OEA: “I am a good observer of others’ emotions”; UOE: “I would always encourage myself to try my best”; ROE: “I am able to control my temper and handle difficulties rationally.” Items were rated on a seven-point Likert scale ranging from 1 (“strongly disagree”) to 7 (“strongly agree”). The scales demonstrated good internal consistency, with Cronbach’s alpha coefficients of 0.88 for SEA, 0.78 for OEA, 0.88 for UOE, and 0.94 for ROE.

CP was assessed using the 3-item scale from Oldham and Cummings (1996) to measure creativity in work settings (e.g., “How original and practical is my work?). A seven-point Likert scale was applied, extending from 1 (“It is not original and practical”) to 7 (“It is original and practical”). The assessment tools demonstrated strong internal consistency (α = 0.92). Slight adaptations were introduced to ensure the items fit the current research design,

Perspective-taking was measured using the PT subscale from the Interpersonal Reactivity Index (IRI) developed by Davis (1983) (e.g., “I believe that there are two sides to every question and try to look at them both”). It included 7 items rated on a five-point Likert scale ranging from 1 (“strongly disagree”) to 5 (“strongly agree”). A robust internal consistency was observed (α = 0.74).

To ensure the validity of the questionnaires originally published in English and applied in Spanish, the standard back-translation technique was employed (Brislin, 1970). Similarly, the cultural adaptation procedure (Beaton et al., 2000) was utilized to ensure that the questionnaire was comprehensible and suitable for the target population. A pilot test with 32 employees at a smaller financial institution was conducted to evaluate the internal consistency and validity of the questionnaires. After the pilot test, small modifications in particular questions were necessary to ensure semantic validity. Common method bias was avoided using Podsakoff et al. (2003) procedure by protecting participants’ anonymity and motivating them to deliver honest responses, as there were no correct or incorrect answers. In this way, evaluation apprehension was reduced, and socially desirable responses were avoided.

### 3.3 Data collection

Initially, Directors of the banks’ Human Resource Departments were contacted and met with to explain the study’s objectives and request their collaboration in data collection. The meetings identified participants for the study working in different departments. The Human Resource Department sent emails explaining the purpose of the study and requesting collaboration to respond to the surveys. Subsequently, the survey was distributed using the drop-and-collect method (Allred and Ross-Davis, 2011). Data were collected between April and May 2024. The informed consent form included in the survey that employees received explained the study’s purpose and scope. Of the 247 questionnaires distributed to invited participants, 224 were completed and deemed valid, yielding a response rate of 90.7%. The population composition was as follows: 45% of respondents were affiliated with the largest bank, 38% with the second-largest, and 17% with the third-largest. This distribution ensured adequate representation and diversity within the population.

### 3.4 Data analyses

Descriptive statistics show that the majority of the participants were female (62%). Most participants (56%) were between 29 and 38 years old, 59% had a university degree, and 57% had 1 to 10 years of work experience (Table 1). Common method bias was assessed using the Harman single-factor test (Podsakoff et al., 2003). Subsequently, exploratory factor analysis was conducted, revealing an explained variance of 40.05%, below the 50% threshold, indicating that common method bias was not a concern.

**TABLE 1:** Descriptive data.

Demographic variable	Category	Frequency	Percent
Gender	Female	139	62%
	Male	85	38%
Age	19–28 years	64	29%
	29–38 years	125	56%
	39–48 years	26	12%
	49–56 years	9	4%
Marital status	Single	109	49%
	Married	90	40%
	Divorced	19	8%
	Other	6	3%
Academic background	University degree	132	59%
	Postgraduate degree	34	15%
	Other	58	26%
Work experience	0–5 years	47	21%
	6–10 years	81	36%
	11–15 years	57	25%
	16–20 years	24	11%
	21–25 years	4	2%
	26–30 years	8	4%
	31–35 years	3	1%

Data were analyzed using SmartPLS 4 software, employing partial least squares structural equation modeling (PLS-SEM). An *a priori* statistical power analysis was conducted using G*Power (Faul et al., 2009). Following PLS-SEM recommendations, the analysis focused on the endogenous construct with the most predictors (five). Assuming a medium effect size (f^2^ = 0.15), a significance level of 0.05, and a statistical power of 0.95, the minimum required sample size was 138 observations. Therefore, the number of participants exceeded the minimum required to detect statistically significant relationships.

**FIGURE 2:**
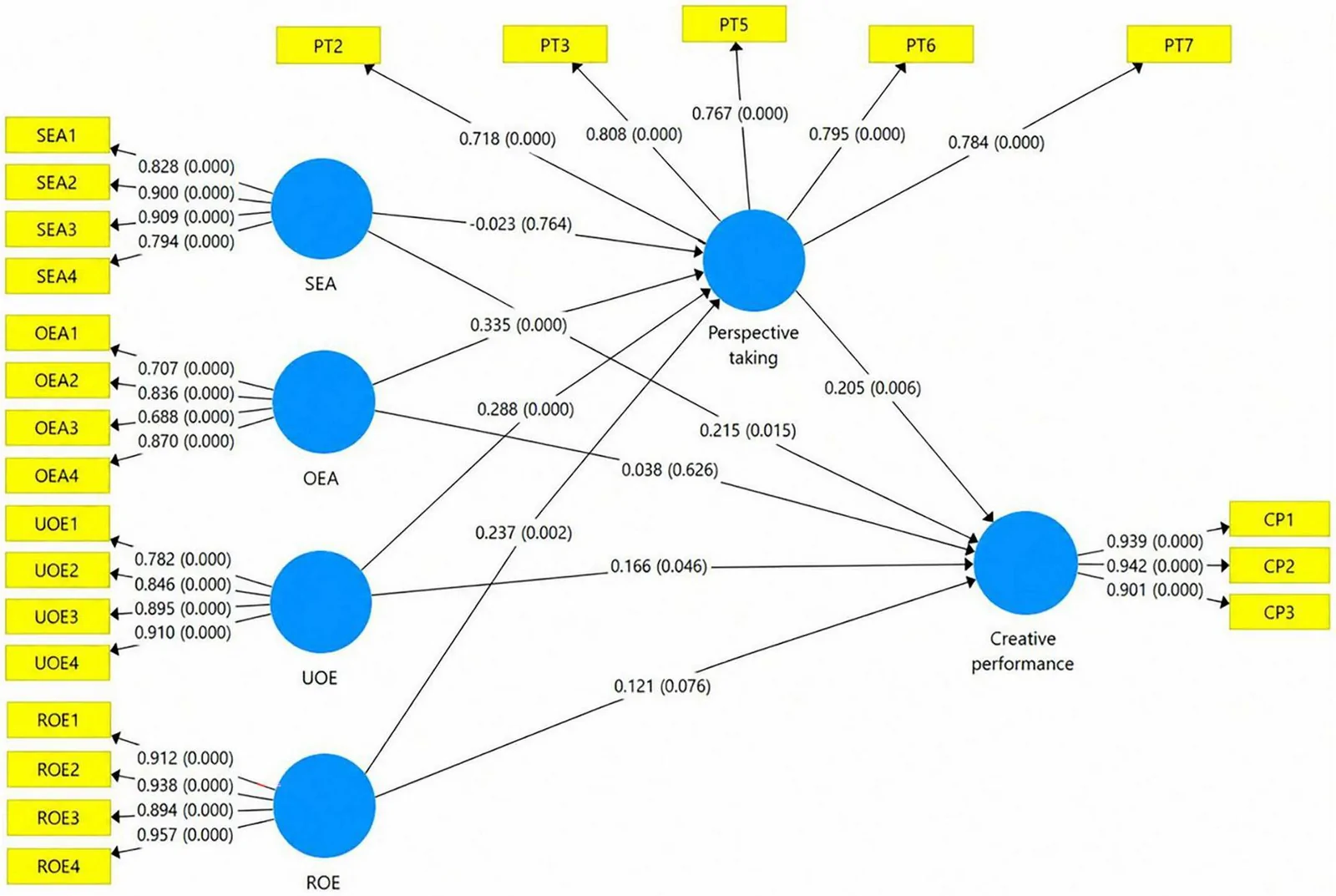
Model estimation. SmartPLS output - first stage.

**FIGURE 3:**
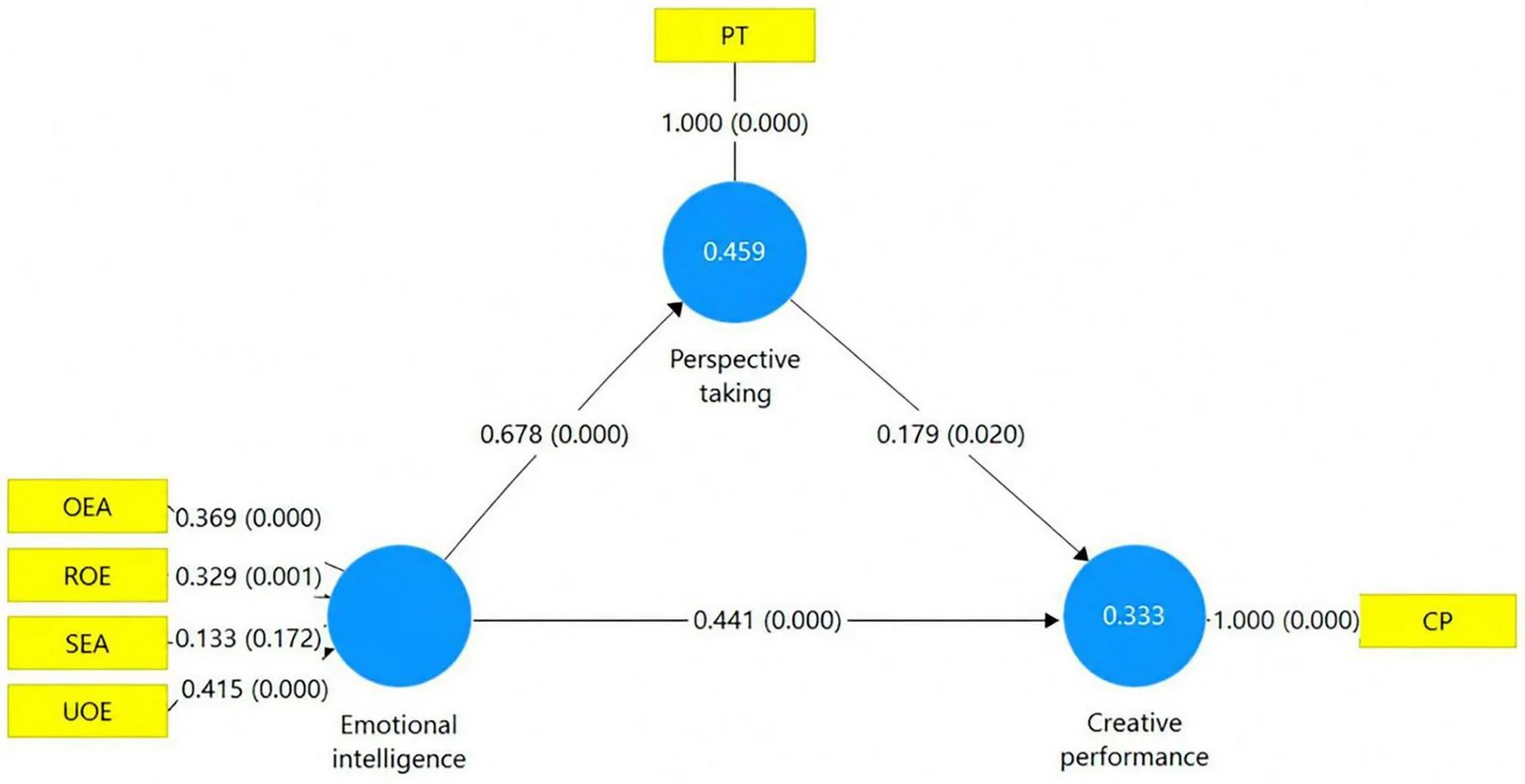
Model estimation. SmartPLS output - second stage.

The specifications of Sarstedt et al. (2019) have been used to estimate higher-order constructs. In this case, emotional intelligence is a second-order formative construct composed of 4 reflective constructs: SEA, OEA, UOE, and ROE. For its estimation, we have implemented the embedded two-stage approach. In the first step, the model was estimated without emotional intelligence (Figure 2). After estimating this model, the latent variable scores for all constructs were saved. In the second step, the structural model with emotional intelligence was estimated (Figure 3). For hypotheses 1, 2, 3, and 4, the path coefficients from the second-step results are analyzed. For the remaining hypotheses, the path coefficients from the first-step results were analyzed. In this way, the same model is used, and all the results were obtained. In other words, we have leveraged the nature of the embedded two-stage approach to estimate higher-order constructs, allowing us to obtain results for all the hypotheses.

## 4 Results

### 4.1 Evaluation of the measurement model

The first step in evaluating the measurement model was to calculate reliability and convergent validity indicators using Hair et al. (2022) considerations. Reflective models must meet the following indicators with their respective thresholds: outer loadings (OL > 0.7), Cronbach’s alpha (CA: 0.70 - 0.90), composite reliability (CR: 0.70–0.90), and average variance extracted (AVE >0.5). Outer loadings >0.708 indicate that the construct explains more than 50% of the indicator’s variance. Higher values for CA and CR indicate greater reliability; a range of 0.70–0.90 is considered satisfactory, while values above 0.95 may be problematic, suggesting undesirable response patterns or redundant items, which reduce construct validity (Diamantopoulos et al., 2012). An AVE value of 0.50 or higher indicates that, on average, the construct explains more than half of the variance of its indicators. On the contrary, an AVE value of less than 0.50 indicates that, on average, more variance remains in the items’ error than in the variance explained by the construct.

The first run of the model (Model 1) identified two items with low loadings: PT1 (0.087) and PT4 (0.025). These items were removed, and the final structural model (Model 2) was run again, confirming item reliability. Internal consistency was also verified by CA and CR values >0.7, and AVE values >0.5 confirmed convergent validity. Table 2 shows these results.

**TABLE 2:** Reliability and convergent validity.

Construct	Model 1				Model 2 (refined model)			
	OL	CA	CR	AVE	OL	CA	CR	AVE
Creative performance		0.919	0.949	0.860		0.919	0.949	0.860
CP1	0.939				0.939			
CP2	0.942				0.942			
CP3	0.901				0.901			
Perspective taking		0.736	0.799	0.430		0.833	0.882	0.601
PT1	0.087				–			
PT2	0.719				0.718			
PT3	0.808				0.808			
PT4	0.025				–			
PT5	0.765				0.767			
PT6	0.794				0.795			
PT7	0.784				0.784			
SEA		0.881	0.918	0.738		0.881	0.918	0.738
SEA1	0.828				0.828			
SEA2	0.900				0.90			
SEA3	0.909				0.909			
SEA4	0.794				0.794			
OEA		0.782	0.860	0.607		0.782	0.860	0.607
OEA1	0.708				0.707			
OEA2	0.837				0.836			
OEA3	0.687				0.688			
OEA4	0.869				0.870			
UOE		0.881	0.919	0.739		0.881	0.919	0.739
UOE1	0.782				0.782			
UOE2	0.847				0.846			
UOE3	0.895				0.895			
UOE4	0.910				0.910			
ROE		0.944	0.960	0.857		0.944	0.96	0.857
ROE1	0.912				0.912			
ROE2	0.939				0.938			
ROE3	0.893				0.894			
ROE4	0.957				0.957			

OL, outer loadings; CA, Cronbach’s alpha; CR, composite reliability; AVE, average variance extracted.

The Fornell-Larcker criterion was applied to confirm the discriminant validity of the latent variables. According to this criterion, the square root of the AVE should exceed the correlation between the constructs (Hair et al., 2022), which was true for these variables (diagonal values > values below the diagonal). The heterotrait-monotrait ratio of correlations (HTMT) was also utilized to confirm discriminant validity. HTMT values should be below the threshold of 0.90 (Henseler et al., 2015). As each value above the diagonal was less than 0.90, discriminant validity was not a concern (Table 3).

**TABLE 3:** Discriminant validity.

Construct	CP	PT	SEA	OEA	UOE	ROE
CP	**0.928**	0.545	0.537	0.457	0.542	0.481
PT	0.478	**0.775**	0.506	0.692	0.65	0.606
SEA	0.484	0.434	**0.859**	0.583	0.677	0.568
OEA	0.391	0.569	0.482	**0.779**	0.543	0.552
UOE	0.491	0.557	0.593	0.457	**0.860**	0.595
ROE	0.454	0.543	0.527	0.48	0.548	**0.925**

CP, creative performance; PT, perspective-taking; SEA, self emotional appraisal; OEA, others emotional appraisal; UOE, use of emotions; ROE, regulation of emotions. Bold diagonal values indicate the square root of the average variance extracted (√AVE).

### 4.2 Evaluation of the structural model

After confirming the validity, reliability, and internal consistency of the measurement model, the next step in PLS-SEM analysis was to evaluate the structural model. Firstly, it was necessary to ensure there were no collinearity issues, which would guarantee that the path coefficients are not biased. To confirm this, the variance inflation factor (VIF) values in the predictor construct must be below 5 or, even better, below 3 (Hair et al., 2022). The present study reported VIF values of 1.85 for both predictive constructs, EI and PT, which eliminated the possibility of substantial collinearity effects in the structural model.

To evaluate the structural model, the explanatory power was calculated using the coefficient of determination (*R*^2^). It reflects the overall impact of the external latent variables on the internal latent variable (Hair et al., 2022). Higher *R*^2^ values mean stronger explanatory power. As a guideline, *R*^2^ values of 0.75, 0.50, and 0.25 are regarded as substantial, moderate, and weak, respectively (Hair et al., 2019). The explanatory power of the variables in this study was considered moderate for both variables: CP (*R*^2^ = 0.333) and PT (*R*^2^ = 0.459). The slight reduction of values for adjusted *R*^2^ (CP adjusted R^2^ = 0.327 and PT adjusted *R*^2^ = 0.457) was an acceptable consequence of increased model complexity resulting from the incorporation of theoretically meaningful predictors.

The effect size indicator for the predictors on the dependent variables, f 2, was analyzed. According to Cohen (1988), the guidelines for evaluating *f ^2^* suggest that values of 0.02, 0.15, and 0.35 correspond to small, medium, and large effects, respectively, of the exogenous latent variable. EI - CP registered *f^2^* = 0.158 (medium effect). EI - PT showed an indicator *f^2^* = 0.850 (large effect). In contrast, PT – CP presented *f^2^* = 0.026 (small effect). These findings suggest that emotional intelligence is the fundamental factor for explaining creative performance in the model, while perspective-taking accounts for a relatively minor portion of the total variance.

The predictive performance of the structural model was assessed using the PLS predict procedure. As shown in Table 4, all *Q*^2^ predict values were greater than zero, ranging from 0.214 to 0.331, indicating that the model exhibits predictive relevance for all endogenous indicators. Furthermore, the PLS model consistently produced lower prediction errors than the linear regression benchmark across all indicators. Specifically, the RMSE and MAE values obtained from the PLS model were lower than those of the LM model for all indicators. According to the guidelines proposed by Shmueli et al. (2019). These results indicate that the PLS model demonstrates medium predictive power, as it outperforms the naïve linear benchmark across all prediction metrics.

**TABLE 4:** Predictive power.

Indicators	*Q*-square	PLS		LM	
		RMSE	MAE	RMSE	MAE
CP1	0.266	1.287	0.990	1.317	1.034
CP2	0.290	1.187	0.898	1.198	0.920
CP3	0.214	1.448	1.166	1.466	1.147
PT2	0.240	0.668	0.523	0.702	0.541
PT3	0.331	0.605	0.490	0.627	0.497
PT5	0.231	0.611	0.500	0.631	0.501
PT6	0.253	0.811	0.655	0.852	0.666
PT7	0.262	0.721	0.584	0.762	0.610

CP, creative performance; PT, perspective-taking; PLS, partial least square model; LM, linear regression model; RMSE, root means square error; MAE, mean absolute error.

Finally, to assess potential endogeneity in the structural model, the Gaussian copula approach was implemented. This method is essential for determining whether the independent variables -Emotional Intelligence and Perspective-Taking- are correlated with the structural error term, which could lead to biased and inconsistent parameter estimates (Hult et al., 2018). The analysis involved three distinct Gaussian copula models to test for endogeneity in EI, PT, and both variables simultaneously. The empirical results demonstrated that the *p*-values for the copula terms (copulaEI and copulaPT) consistently exceed the standard 0.05 significance threshold. Specifically, the *p*-values for copulaEI ranged from 0.6259 to 0.6853, and for copulaPT from 0.7835 to 0.9571. Given that the copula terms are not statistically significant, the null hypothesis of no endogeneity cannot be rejected. This evidence confirms that neither Emotional Intelligence nor Perspective-Taking is affected by endogeneity in this model. Therefore, the path coefficients and results derived from the original structural model are considered robust and consistent, providing confidence that the identified influences on creative performance are not biased by omitted variables or other systematic errors.

### 4.3 Hypotheses testing

The significance of the structural model relationships was established through analysis of the path coefficients. Path coefficients (β) close to +1 indicate strong positive relationships; they should be at least 0.1 to reflect a meaningful effect within the model (Hair et al., 2022). To support the hypothesized relationships, these indicators were calculated, and standard errors were obtained via bootstrapping with 10,000 resamples. A significance level of 0.05 was used to support research hypotheses (Table 5). Regarding the primary model, the results were as follows: H1 held that EI positively relates to CP. This hypothesis was supported, confirming that EI positively affects CP (β = 0.441, *p* < 0.001). H2 was also supported; EI has a strong positive effect on PT (β = 0.678, *p* < 0.001). The results showed that PT has a positive effect on CP, confirming H3 (β = 0.179, *p* = 0.020). Regarding the mediating effect, the results supported H4, confirming that PT mediates the relationship between EI and CP (β = 0.121, *p* = 0.024). The model presented a complementary mediation, which, according to Zhao et al. (2010), occurs when the mediated effect (EI-PT-CP) and the direct effect (EI-CP) are present and both point in the same direction.

**TABLE 5:** Bootstrapping results.

Relationship	*B*	Standard deviation	*t*-Statistic	*P*-value	IC 95%	Hypothesis decision
Second stage						
EI → PT	0.678	0.056	12.124	***	[0.560; 0.775]	Supported
PT → CP	0.179	0.077	2.322	0.020	[0.016; 0.316]	Supported
EI → CP	0.441	0.074	5.921	***	[0.299; 0.594]	Supported
EI → PT CP	0.121	0.054	2.253	0.024	[0.011; 0.221]	Supported
First stage						
SEA → PT	−0.023	0.076	0.3	0.764	[−0.173; 0.127]	Not supported
OEA → PT	0.335	0.062	5.395	***	[0.213; 0.455]	Supported
UOE → PT	0.288	0.078	3.694	***	[0.128; 0.434]	Supported
ROE → PT	0.237	0.078	3.03	0.002	[0.09; 0.395]	Supported
SEA → CP	0.215	0.089	2.426	0.015	[0.037; 0.383]	Supported
OEA → CP	0.038	0.077	0.488	0.626	[−0.115; 0.185]	Not supported
UOE → CP	0.166	0.083	1.996	0.046	[0.001; 0.331]	Supported
ROE → CP	0.121	0.068	1.772	0.076	[−0.001; 0.265]	Not supported
SEA → PT → CP	−0.005	0.017	0.282	0.778	[−0.038; 0.029]	Not supported
OEA → PT → CP	0.069	0.028	2.454	0.014	[0.019; 0.128]	Supported
UOE → PT → CP	0.059	0.029	2.052	0.040	[0.012; 0.123]	Supported
ROE → PT → CP	0.048	0.023	2.110	0.035	[0.011; 0.099]	Supported

***p-value < 0.001; IC = 95% confidence interval.

The relationships of the secondary model were also evaluated (Table 5) and showed that four of the 12 hypotheses were not supported. The results indicated that H1a, which posited a positive relationship between SEA and PT, was not confirmed. Similarly, OEA and ROE have an insignificant effect on CP; consequently, H3b and H3d were not supported. Finally, H4a proposed a mediation relationship of PT between SEA and CP, which was not supported.

## 5 Discussion

Currently, creativity research focuses on applied areas. Being creative in organizations is one of the research topics that stands out (De-Marchis and Shchebetenko, 2023), particularly in knowledge-intensive sectors, such as banks (Nguyen and Nguyen, 2024). Understanding the creative processes of employees in banking institutions has attracted considerable interest, particularly in developing countries (Khan and Zulfiqar, 2024). The results of the present study confirmed all the primary model hypotheses and eight of the twelve hypotheses of the secondary model. The results showed that EI has a strong positive association with PT (H1), consistent with Pongrac et al. (2019) and Shi and Du (2020), as adopting another’s perspective evokes numerous emotions (Brügge-Feldhake et al., 2024). The connection between EI and cognitive empathy creates synergy, enabling employees with high levels of EI to be more open to observing, socializing, and communicating with others. All dimensions of emotional intelligence, except SEA, were positively associated with PT. This finding contrasts with that of Rieffe and Camodeca (2016), who suggested that greater attention to one’s own emotions promotes the development of more sophisticated empathic abilities. A possible explanation is that PT is inherently other-oriented, requiring individuals to move beyond their own perspective to understand another person’s thoughts, emotions, and intentions. In contrast, SEA primarily reflects self-awareness by enhancing individuals’ understanding of their own emotional states; however, it does not necessarily foster the cognitive flexibility or outward focus required to appreciate others’ perspectives. Furthermore, because an individual’s emotional state dictates the extent to which they prioritize their own viewpoint during PT, when negative emotions are present, they can significantly intensify their self-focus (Mor and Winquist, 2002), which means that a person with high self-emotional appraisal (SEA) who is acutely aware of their own distress may experience a reduction in the cognitive bandwidth required to move beyond their internal reference point. Consequently, such intense self-attunement can impede the cognitive flexibility and external orientation necessary to comprehend others’ perspectives accurately. By comparison, OEA, UOE, and ROE encompass interpersonal and adaptive competencies that more directly facilitate the understanding of others, thereby contributing more strongly to perspective-taking abilities.

The confirmation of H2, which posits a strong positive relationship between PT and CP, demonstrates that understanding the perspectives of internal and external clients will contribute to the generation of useful creative outcomes. These results are consistent with the findings of Kung and Chao (2019) and Nadiia et al. (2019). The current study supports the idea that the creative process is affected by empathy, as more empathic individuals tend to perform better on creativity-related tasks, such as divergent thinking (Anderson et al., 2023; Chen et al., 2021). In this sense, it is relevant to consider that excessive empathy can impede everyday creativity (Form and Kaernbach, 2018).

H3 was supported, demonstrating that EI has a strong positive relationship with the CP of banks’ employees. This finding is consistent with DCTC that emphasizes the importance of emotions and social interaction for creativity and proposes that creativity is influenced not only by domain expertise and creative-thinking skills but also by individual characteristics that support intrinsic motivation and adaptive functioning. Emotional intelligence may foster these conditions by enabling employees to manage emotional demands, remain engaged in problem-solving, and persist when encountering obstacles, which implies that the creative potential of the emotionally intelligent employee may be triggered. These results are consistent with previous studies reporting positive associations between emotional intelligence and creativity (e.g., Darvishmotevali et al., 2018; Fujii, 2025; Kasuma and Rusdi, 2024; Stawicki et al., 2022; Shafait and Huang, 2024). These studies argue that emotionally intelligent individuals are better able to transform emotional experiences into cognitive and motivational resources that enhance idea generation and creative problem solving. The present study extends this evidence by demonstrating that the relationship also holds in the Ecuadorian banking sector, a context characterized by stringent regulatory requirements, standardized procedures, and increasing pressure for innovation through digital transformation and customer-centered services. In this context, even negative emotions, such as loneliness, considered to harm CP (Firoz and Chaudhary, 2022), may be taken advantage of since EI is linked to creativity from both positive and negative emotions (Leung et al., 2021; Liu et al., 2022).

However, this study reveals that not all EI subdimensions have a positive or significant relationship with CP. SEA and UOE were positively associated with employees’ creativity, consistent with Leung et al. (2021), whereas the hypotheses linking OEA and ROE were not supported. Contrary to expectations, Regulation of Emotion did not exhibit a significant direct effect on creative performance, inconsistent with Carmeli et al. (2013). A possible explanation is that emotion regulation primarily enables employees to maintain emotional stability and cope effectively with workplace stress. However, these capabilities alone may be insufficient to promote creative behavior. According to the Componential Theory of Creativity (Amabile and Pratt, 2016), creativity depends largely on intrinsic motivation, domain-relevant expertise, and creative-thinking skills. Therefore, although emotion regulation may provide a favorable psychological foundation, its influence may operate indirectly through cognitive and interpersonal mechanisms, such as perspective-taking, rather than directly affecting creative performance. Furthermore, the highly regulated banking environment may limit employees’ opportunities to translate emotional self-regulation into observable creative outcomes, thereby attenuating the direct relationship.

Similarly, the insignificant connection between OEA and CP suggests that the mere ability to recognize and understand others’ emotions is insufficient to independently trigger the generation of novel ideas; however, a significant indirect relationship exists through the mediation of PT. This confirms that appraising others’ emotions translates into creative performance only when it empowers the individual to actively adopt the viewpoints of internal or external clients, thereby channeling social insights into innovative solutions that are both original and practically useful.

These findings suggest that creative performance is more strongly influenced by individuals’ capacity to effectively utilize emotions and accurately appraise their own emotional states than by their ability to regulate emotions or perceive others’ emotions. Moreover, excessive emotional regulation may potentially constrain the spontaneity, openness, and cognitive experimentation that are often essential for creativity.

Finally, the mediating effect of PT on the relationship between EI and CP, supported by H4, provides a powerful explanation of how internal emotional abilities translate into practical, innovative outcomes and shows that the capacity to deal with emotions fosters an understanding of other people’s perspectives that encourages them to explore new ideas and solutions. The confirmation of this mediation supports Bandura’s SCT, which posits that human behavior results from a dynamic interplay among personal factors, environmental influences, and cognitive processes.

The mediating role of PT was also observed in the relationship between three subdimensions of EI and CP. Perspective-taking plays an important role when OEA, UOE, and ROE unleash creativity, but it is not relevant between SEA and CP. The failure to support the SEA-PT-CP mediation suggests that understanding one’s own feelings is a distinct intrapersonal asset that directly triggers creativity. In contrast, interpersonal navigation (PT) is required when social insights are used to unleash innovative solutions. In this context, PT can be viewed as a mediator that enhances social learning and problem-solving, thereby boosting creative performance. EI and PT are competencies that enable harmonious, effective workplace interactions, fostering a positive environment for the emergence of creative products that are not only original and useful but also appropriate and beneficial to all parties involved. These findings reveal two complementary yet distinct emotional routes to creativity: a self-oriented and direct pathway, and a socially oriented one that operates through perspective taking.

The study’s findings are significantly shaped by Ecuador’s strong collectivist culture, which is known to have the highest PT score globally. This cultural background, which emphasizes interdependence and an orientation toward others, explains why perspective-taking serves as a vital socially oriented bridge that channels interpersonal emotional competencies into creative performance. Specifically, the results show that while recognizing others’ emotions or regulating one’s own does not directly trigger creativity, these skills become effective when they empower individuals to actively adopt others’ viewpoints, a trait highly valued in collectivist societies. Conversely, in non-collectivist or individualistic cultures that prioritize independence and focus primarily on the self, research might yield different results; such contexts might reveal more direct effects of self-oriented emotional traits on creativity or a less central role for perspective-taking as a mediating mechanism.

### 5.1 Managerial implications

The results of this study have practical implications for companies and their HR Directors. Although data were collected from middle-level managers, the findings provide insights that extend beyond the individual level, offering practical implications for organizations seeking to enhance creativity by developing emotional intelligence and perspective-taking capabilities. The literature suggests that organizations prioritize the individual traits of their middle managers (Moccia et al., 2026). In line with this perspective, the present study recommends that HR Directors evaluate applicants’ EI and PT levels during talent selection and give preference to those with higher scores. Given that EI and empathy training programs are effective (Iyanda et al., 2024; Jian et al., 2025) employees should be trained by the company to boost EI (Mahbub and Barhate, 2025), especially in the post-pandemic scenario, where a pronounced and persistent reduction in emotional intelligence has been identified at a global level between 2019 and 2024 (Freedman et al., 2025). Likewise, empathy training through experimental activities, such as mindfulness meditation, can strengthen emotional self-efficacy and enhance an individual’s confidence in their emotional and interpersonal abilities (Balwant, 2026). This kind of instruction must be carried out to strengthen individual employees’ creativity, which is difficult to imitate, rather than relying on procedural creativity, which is easy for competitors to emulate (Khan and Zulfiqar, 2024).

### 5.2 Theoretical implications

This study confirmed Bandura’s SCT in a new context using a population of middle managers from the banking sector. It also confirmed the DCTC (Amabile and Pratt, 2016), and the AMEI (Mayer and Salovey, 1997); when applied in an emerging economy such as Ecuador It extends the applicability and relevance of these theories beyond the developed world. It also contributes to the CP literature by proposing a structural model with moderate explanatory power and relevant predictive power for understanding how EI and PT may promote CP in an unexplored sector. To our knowledge, this is the first time the subdimensions of EI, as proposed by Wong and Law (2002), have been studied in an organizational context. Additionally, it was conducted in a geographical context and an industrial sector where such studies are scarce. Similarly, the multidimensional approach to measuring EI raises new research questions.

### 5.3 Social implications

Boosting employees’ creativity by strengthening their EI and PT could make a difference in the way organizations interact with their customers. Employees’ creative efforts may result in friendlier products and simplified processes for those who still face limitations in accessing financial services, such as the elderly, SMEs, or new entrepreneurs. Furthermore, PT influence during the development of creative outcomes could encourage creative individuals to develop greater moral maturity. Solutions designed under morally mature considerations will not sacrifice ethical principles (Hui et al., 2022). This positive creativity contributes to the objectives of sustainable bank models, such as ethical banking, which not only seeks economic benefits but also guarantees transparency and social benefits (Valls Martínez et al., 2021).

### 5.4 Limitations and recommendations for future research directions

These findings should be interpreted with the following limitations in mind. The cross-sectional nature of the study design limits these results to a specific moment in time. A longitudinal study could demonstrate the stability of the cause-and-effect relationships among the variables. The data were collected in large banks in Ecuador, which limits generalizability to other sectors such as manufacturing or IT. Similarly, the study may not reflect the experiences and cognitive demands of employees with different functions, such as frontline employees or top-level executives. Finally, it may yield different results if replicated in individualistic cultures that prioritize independence and self-focus over interdependent social navigation. Future research should aim to test this model across diverse industries, hierarchical levels, and national settings to enhance the external validity of these findings. It should also focus on understanding the relationships between EI subdimensions and CP with other moderating or mediating variables. Similarly, the possibility of generating original and useful achievements triggered by negative emotions deserves more research. In the same way, the analysis of the negative effect of excessive empathy as a mediator in this relationship, as well as of other models that deter negative and malevolent creativity, is recommended.

## 6 Conclusion

The results of this study suggest that emotional intelligence is significantly associated with creative performance among mid-level managers at large private banks in Ecuador. A key finding indicates that perspective-taking functions as a complementary mediator in this relationship, a role that appears particularly vital within the Ecuadorian collectivist context, where understanding others’ viewpoints is a highly prioritized social value. The evidence indicates that for this sample, emotional competencies allow individuals to navigate social dynamics more effectively, thereby facilitating the translation of emotional information into practical and innovative workplace solutions. Cultivating these interpersonal skills may not only bolster individual creativity, potentially offering a competitive advantage that is difficult for rivals to replicate, but also appear to encourage moral maturity. Ultimately, integrating these “soft skills” into the banking sector may advance sustainable and ethical banking models that prioritize transparency and social benefits alongside organizational objectives.

## Data Availability

The raw data supporting the conclusions of this article will be made available by the authors, without undue reservation.
